# Editorial: Distal, medium vessel occlusion stroke: epidemiology, diagnostics, treatment options, and outcome prediction

**DOI:** 10.3389/fstro.2026.1967379

**Published:** 2026-09-15

**Authors:** Gabriel Broocks, Panagiotis Papanagiotou, Leonard L. L. Yeo, Anil Gopinathan, Eytan Raz, Lukas Meyer

**Affiliations:** 1 Department of Neuroradiology, Helios Medical Center Schwerin, University Campus of MSH Medical School Hamburg, Schwerin, Germany; 2 Bremen-Mitte Clinic, Bremen, Germany; 3 Division of Neurology, National University Health System, Singapore, Singapore; 4 National University of Singapore, Singapore, Singapore; 5 Department of Diagnostic Imaging, National University Health System, Singapore, Singapore; 6 Department of Radiology, New York University, New York, NY, United States; 7 Department of Neuroradiology, University Medical Center Hamburg-Eppendorf, Hamburg, Germany

**Keywords:** aspiration [MeSH], DMVOs, stent retriever (SR), stroke, thrombectomy

Mechanical thrombectomy has transformed the treatment of acute ischemic stroke caused by large-vessel occlusion. Advances in low-profile aspiration catheters, microcatheters, and stent retrievers subsequently rendered increasingly distal arterial territories technically accessible, generating considerable interest in endovascular treatment (EVT) for distal or medium-vessel occlusion (DMVO) stroke. DMVO, however, is not a single disease entity. Occlusions differ in vessel diameter, anatomical segment, eloquence of the affected territory, collateral supply, clinical deficit severity, thrombus origin, and procedural accessibility. Primary DMVOs must also be distinguished from secondary distal occlusions that arise through spontaneous or treatment-related thrombus migration after an initially proximal occlusion.

This Research Topic was conceived during a period of rapid technical expansion and growing observational evidence. Since then, the field has entered a phase of evidence-based recalibration. Three randomized trials—DISTAL (Psychogios et al., 2025), ESCAPE-MeVO (Goyal et al., 2025), and the early-terminated DISCOUNT trial (Clarençon et al., 2026)—did not demonstrate a clinical benefit of routine thrombectomy in broadly selected DMVO populations and raised concerns regarding procedure-related harm, including symptomatic intracranial hemorrhage and, in some cohorts, mortality signals. In contrast, ORIENTAL-MeVO reported that among Chinese patients with medium-vessel occlusion and moderate-to-severe deficits (National Institutes of Health Stroke Scale [NIHSS] score ≥6), EVT increased 90-day functional independence from 46.6% to 58.6% (adjusted rate ratio, 1.24; 95% confidence interval [CI], 1.07–1.44), although symptomatic intracranial hemorrhage occurred in 4.7% vs. 2.2% (Hu et al., 2026). This divergent result underscores the influence of trial population, occlusion definition, thrombolysis use, and procedural strategy and argues against treating DMVO as a single therapeutic indication.

Neutral treatment effects should not be mistaken for a benign natural history. Across contemporary randomized control groups, 32%−53% of medically managed patients did not achieve functional independence at 90 days; this proportion exceeded 45% in DISTAL (45.3%) and ORIENTAL-MeVO (53.4%) (Psychogios et al., 2025; Goyal et al., 2025; Clarençon et al., 2026; Hu et al., 2026). These control arms are among the most robust prospective repositories of outcome data under contemporary medical care and show that DMVO frequently results in death or persistent dependency. This observation does not justify indiscriminate thrombectomy: technical accessibility alone does not establish a treatment indication, and expected benefit must exceed procedural risk. It does, however, show that both routine EVT as tested and medical treatment alone remain unsatisfactory for many patients, reinforcing the need for better selection and safer or more effective reperfusion strategies.

The trials, therefore, sharpen rather than settle the central question of patient selection, particularly for M2 occlusions, whose anatomical and functional definitions remain heterogeneous (Mehta et al., 2025). Proximal or dominant M2 branches lie closer to classical large-vessel-occlusion evidence than truly distal M3, A3, or P3 occlusions. Observational data suggest that any benefit of successful reperfusion may concentrate in specific phenotypes: in M2 stroke, the association between recanalization and 90-day functional independence was larger at higher baseline stroke severity (Broocks et al., 2026), and penumbral imaging identified subgroups defined by larger hypoperfusion or penumbral volumes in which reperfusion was associated with better outcome (Broocks et al., 2025). These hypothesis-generating findings support imaging- and severity-informed selection rather than a purely anatomical indication.

Several contributions to this Research Topic address the balance between reperfusion efficacy and vessel safety. The systematic review and meta-analysis by Loh et al. found that combined stent-retriever and aspiration techniques were associated with higher first-pass reperfusion and lower symptomatic intracranial hemorrhage than single-device approaches, although final functional outcomes were similar; these retrospective data concern technique selection among treated patients and do not establish superiority of thrombectomy over medical treatment. The ONE-SEG technique of Hamada et al., combining limited deployment of the distal basket segment of a stent retriever with aspiration, achieved high reperfusion rates in a small M2 cohort, with outcomes differing substantially between primary and secondary M2 occlusions. Complementing this, Ishiguro et al. linked reduced radial and axial device forces to less vessel deformation on bench testing and a lower frequency of post-procedural subarachnoid hemorrhage. When the expected absolute benefit is small, such reductions in procedural trauma may be decisive.

Newer low-profile devices may alter this procedural risk–benefit balance, although their clinical relevance remains uncertain. In a retrospective series, the small-bore Red43 distal aspiration catheter showed high target-vessel trackability and reperfusion rates without symptomatic intracranial hemorrhage, perforation, or dissection (Goh et al., 2025). For stent-retriever thrombectomy, the randomized DISTALS trial specifically evaluated the radially adjustable low-profile Tigertriever 13 and reported favorable technical efficacy and safety; however, these device-specific angiographic findings should not be equated with established functional benefit (Saver et al., 2026). A complementary strategy is local pharmacological reperfusion. The RESCUE-TNK trial was designed to evaluate intra-arterial tenecteplase for primary or secondary medium-vessel occlusions considered unsuitable for mechanical clot retrieval (Hu et al., 2023). These developments support comparative evaluation of device- and drug-specific reperfusion strategies using clinical rather than purely angiographic endpoints.

Anatomical and clinical heterogeneity is equally central. In an EVT-treated cohort of isolated posterior cerebral artery occlusions, van Landeghem et al. observed that P1 occlusions produced more severe and broader deficits, whereas P2 occlusions more often caused visual syndromes that may be underrepresented by the NIHSS; deep infarction remained common despite angiographic reperfusion. The case series by Xu and Yang illustrates the diagnostic complexity of bilateral paramedian thalamic infarction and the artery of Percheron, in which a unilateral fetal-type posterior cerebral artery may serve as an anatomical clue and treatment targeted upstream large-vessel pathology rather than the perforator itself.

The Research Topic also examines markers of adverse outcome after reperfusion. The meta-analysis by Wu et al. associated early venous filling during thrombectomy with higher intracranial hemorrhage risk and lower functional independence—a potentially useful real-time warning sign that still requires standardized definition and prospective validation.

The next phase of DMVO research should, therefore, move beyond a binary debate over whether distal thrombectomy is safe and effective. Future studies should identify treatment-responsive phenotypes by integrating disabling deficit, eloquent tissue involvement, occlusion segment, collateral and perfusion status, thrombus characteristics, procedural accessibility, and predicted device-related risk, while separating primary from secondary DMVO. Outcome measures should capture vision, language, cognition, fine motor function, and quality of life in addition to the modified Rankin Scale. The neutral randomized trials appropriately challenge indiscriminate treatment; together with the positive ORIENTAL-MeVO signal, they sharpen two essential questions: which carefully selected patients can achieve a meaningful net benefit from rapid complete reperfusion and how future trials should be designed to identify patients most likely to benefit from EVT.
